# A nursing perspective on bronchoscopic intervention strategies for children with macrolide-unresponsive *Mycoplasma pneumoniae* pneumonia

**DOI:** 10.3389/fmed.2026.1878549

**Published:** 2026-08-11

**Authors:** Li-ping Bai, Jiu-mei Wang, Li-na Sun, Jia-rui Yin, Feng-ge Gao, He-xuan Wang, Xiao-na Zhang

**Affiliations:** 1 Department of Pediatrics, Hongqi Hospital Affiliated to Mudanjiang Medical University, Mudanjiang, China; 2 Department of Dermatology, Hongqi Hospital Affiliated to Mudanjiang Medical University, Mudanjiang, China; 3 The First Clinical College, China Medical University, Shenyang, China

**Keywords:** bronchoscopic intervention, immune modulation, macrolide-unresponsive *Mycoplasma pneumoniae* pneumonia, obliterative bronchiolitis, plastic bronchitis, therapeutic time window

## Abstract

Macrolide-unresponsive *Mycoplasma pneumoniae* pneumonia has evolved from a benign, self-limiting infection into a condition capable of progressing to plastic bronchitis and obliterative bronchiolitis. Current guideline-based strategies remain anchored to a reactive logic of antibiotic switching and deferred intervention, thereby missing a critical therapeutic window before irreversible airway remodeling begins. This perspective article proposes a paradigm shift that reconceptualizes bronchoscopy not as a salvage drainage tool, but as a temporally sensitive vehicle for a triadic strategy of immune modulation, microenvironmental re-equilibration, and structural protection. We delineate a three-dimensional stratified interventional framework comprising: early identification of a golden intervention window informed by biomarkers and imaging; modular, phenotype-guided techniques that prioritize mucosal preservation; and peri-interventional management that couples systemic immunomodulation with respiratory mechanics protection. We further identify key translational bottlenecks, including the absence of standardized protocols, reliance on surrogate endpoints rather than preservation of distal airway function, and unresolved safety boundaries in young children. Addressing these gaps through prospective, evidence-generating research is essential to establish this integrated strategy as the new standard of care.

## 1 Introduction

### 1.1 Global epidemiological rise and clinical challenges

*Mycoplasma pneumoniae* pneumonia (MPP) was long regarded as a benign, self-limiting infection (1). However, the global dissemination of macrolide-resistant strains over the past two decades has fundamentally altered its clinical trajectory (2–4). Resistant isolates provoke a dysregulated, Th2-polarized host inflammatory cascade driven by persistent antigenic stimulation following initial macrolide failure, rather than by enhanced intrinsic virulence (1, 4). Affected children consequently develop persistent high fever and intractable cough, progressing rapidly to segmental or lobar consolidation, often complicated by pleural effusion, necrotizing pneumonia, or obliterative bronchiolitis (3, 5). This evolution underscores an urgent need to broaden clinical focus beyond pathogen eradication alone, toward the aberrant immune response and its tissue-damage sequelae (1).

Current definitions of “refractory” disease, anchored to persistent fever beyond 72 h of therapy with radiographic progression, carry substantial temporal lag and diagnostic ambiguity (6–8). Fever, C-reactive protein kinetics, and imaging findings frequently evolve discordantly, trapping clinicians in an indeterminate zone where irreversible mucosal injury may already be underway before criteria are formally met (7–9). This exposes a fundamental weakness in monitoring strategies reliant solely on sequential observation of defervescence and antibiotic failure (6, 8, 9).

### 1.2 Limitations of current guidelines

Prevailing guideline-based decision pathways adopt a linear causal model: pathogen resistance prompts a switch to second-line antibiotics, followed by an observation period for therapeutic effect (8, 10). When defervescence remains inadequate or imaging worsens, the algorithm merely recommends that corticosteroids or bronchoscopic intervention be “considered,” without stratified criteria for timing, duration, or efficacy assessment (8, 10). This reactive, rescue-based strategy presumes a unidirectional and inherently reversible disease course, overlooking the critical pathophysiological reality that once tenacious mucus plugs and fibrinous exudates obstruct the airway lumen, epithelial necrosis, basement membrane denudation, and fibrotic organization are already initiated (8, 11). Even if subsequent anti-inflammatory therapy suppresses the ongoing inflammatory process, the remodeling cascade can still converge inexorably toward obliterative bronchiolitis (8, 12). In this paradigm, bronchoscopy is relegated to a mechanical drainage tool, rather than being positioned as the central fulcrum of integrated therapeutic decision-making.

### 1.3 A conceptual framework

**FIGURE 1:**
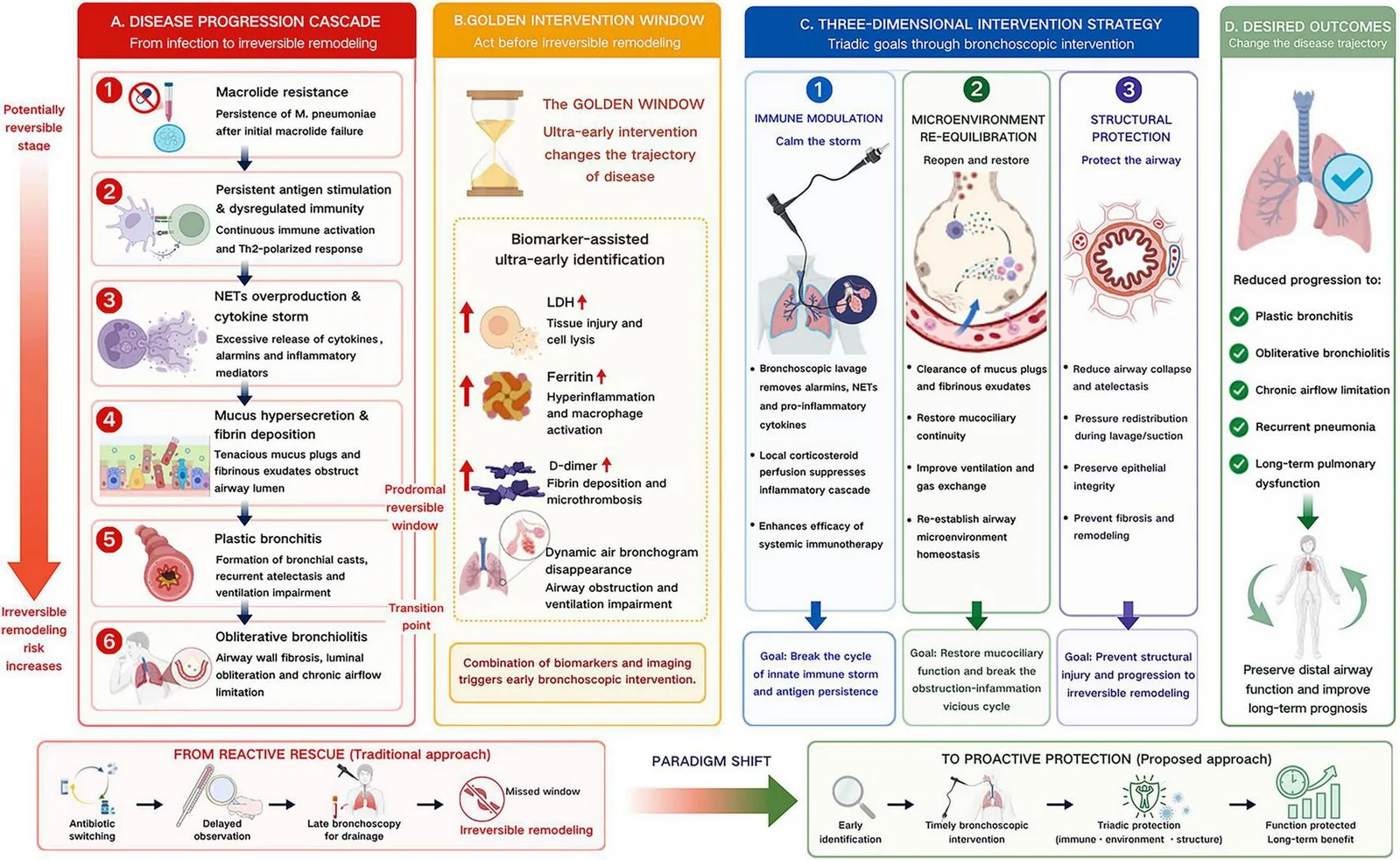
Conceptual framework of temporally stratified bronchoscopic intervention in macrolide-unresponsive *Mycoplasme pneumoniae pneumonia.*

Confronting these impasses, we argue for a fundamental reconceptualization of bronchoscopic intervention, elevating it from a remedial aspiration procedure to a temporally sensitive vehicle driving a triadic strategy of immune modulation, microenvironmental re-equilibration, and structural remodeling (12, 13). First, by promptly clearing mucus plugs rich in alarmins, neutrophil extracellular traps, and pro-inflammatory cytokines, it directly attenuates the innate immune storm and removes local barriers that blunt the effects of systemic corticosteroids (13). Second, lavage and meticulous plug debridement restore mucociliary continuity, breaking the self-perpetuating cycle of obstruction, inflammation, and failed epithelial regeneration (14). Third, early relief of airway obstruction mitigates heterogeneous transpulmonary pressure distribution, preventing the paradoxical coexistence of hyperinflation and atelectasis, thereby preserving alveolar-capillary unit integrity and halting progression toward fibrotic pathology (11). We therefore postulate that a treatment framework centered on optimally timed, early bronchoscopic intervention—integrating immunomodulation with mechanical protection—constitutes the requisite paradigm shift for arresting the transition to chronic irreversible damage and improving long-term outcomes (Figure 1).

## 2 Theoretical reconstitution: novel pathophysiological foundations for bronchoscopic intervention—beyond mucus plug clearance

### 2.1 Reappraising core pathological mechanisms of macrolide unresponsiveness

The pathogenesis of macrolide-unresponsive MPP (MUMPP) extends well beyond simple antimicrobial resistance. Persistent microbial carriage triggers a dysregulated host inflammatory response that constitutes the central driver of disease progression (5, 15). Within this cascade, excessive formation of neutrophil extracellular traps (NETs) plays a pivotal bridging role (5, 16). The chromatin meshwork of NETs not only ensnares pathogens but also directly injures the airway epithelium through its histone and protease constituents, exposing basement membrane collagen and thereby activating the coagulation cascade with subsequent fibrin deposition (15, 16). Simultaneously, NETs-derived alarmins such as high-mobility group box 1 (HMGB1) drive mucin gene overexpression via Toll-like receptor signaling, promoting MUC5AC-dominant mucus hypersecretion (16). This fibrin-rich, hyperviscous secretion resists mucociliary clearance and instead becomes a reservoir for inflammatory mediators, establishing a self-perpetuating cycle of “NETs formation—epithelial injury—mucus hypersecretion—stasis—secondary inflammation” (15, 17). This cycle perpetuates disease progression even after antibiotics have cleared the pathogen.

Plastic bronchitis, traditionally regarded as a rare complication, is now recognized as substantially underdiagnosed in this setting, based on evidence from high-resolution computed tomography and direct bronchoscopic visualization (18). Early in the disease course, fibrinous exudates and mucin interweave to form partially consolidated “pre-cast” material within the subsegmental bronchi, causing partial airway obstruction (18, 19). Since classic manifestations such as expectorated bronchial casts or the “finger-in-glove” radiographic sign have not yet emerged at this stage, the diagnosis is frequently missed (18, 19). Such delayed recognition allows initially reversible endoluminal occlusion to evolve into organized, adherent casts that require more complex retrieval (19).

We further posit the “prodromal window” hypothesis of obliterative bronchiolitis, which contends that the transition from macrolide-unresponsive pneumonia to obliterative bronchiolitis is not an uninterrupted, inevitable progression. Rather, a recognizable therapeutic window exists. Before the bronchiolar epithelium undergoes irreversible denudation and granulation tissue obliterates the lumen, the pathological process remains predominantly reversible, confined to inflammatory edema, mucus plugging, and fibrin deposition (20, 21). During this prodromal phase, timely removal of intraluminal obstructive material can avert epithelial-mesenchymal transition and fibroblast activation, thereby forestalling the development of obliterative bronchiolitis (20, 21). This hypothesis provides a crucial temporal rationale for early bronchoscopic intervention.

### 2.2 Three novel therapeutic targets for bronchoscopic intervention

Bronchoscopic intervention offers three mechanistically distinct yet synergistic therapeutic targets. The first operates at the level of respiratory mechanics (22, 23). By directly evacuating hyperviscous secretions and fibrinous casts from the airway lumen, the procedure immediately reduces airway resistance, respiratory muscle workload, and the heterogeneous distribution of transpulmonary pressure (22, 24). This mechanical decompression disrupts the stress-induced tissue injury caused by obstructive ventilatory impairment and curtails the persistent release of inflammatory mediators (22, 24).

The second target addresses the airway mucosal immune microenvironment (25). Retained mucus plugs function not merely as physical obstructions but as toxic reservoirs enriched with NETs components, HMGB1, and multiple pro-inflammatory cytokines (23, 25). These mediators continuously activate airway epithelial and resident immune cells, sustaining a localized innate immune storm (25). Bronchoscopic lavage physically removes this inflammatory reservoir, thereby creating conditions permissive for subsequent anti-inflammatory pharmacotherapy and the restoration of local immune homeostasis (24).

The third target concerns structural protection of the terminal airways (23). When severe mucus plug obstruction leads to distal air absorption and alveolar collapse, the loss of parenchymal tethering predisposes the bronchiole wall to concentric collapse and apposition (23). This provides a structural substrate conducive to intraluminal granulation tissue in growth (26). Early restoration of airway patency therefore not only improves oxygenation but, more fundamentally, preserves the normal anatomical architecture of the bronchiole and prevents irreversible obliterative lesions (23, 26). Operating in concert, these three targets substantiate the role of bronchoscopy as a pathogenesis-directed intervention that transcends the traditional paradigm of simple mucus plug clearance (23, 26, 27).

## 3 A novel strategic framework: a three-dimensional stratified interventional system based on the therapeutic time window

### 3.1 Time-window stratification: from delayed, passive response to early, active intervention

The conventional 72-h threshold for identifying macrolide unresponsiveness should be redefined as the starting point of a critical intervention window, not the endpoint of a waiting period (23, 28). Pathophysiological evidence indicates that during these initial 72 h of persistent fever and radiographic progression, fibrinous airway exudates remain in a reversible, gel-like phase, before extensive subepithelial fibrosis and irreversible wall remodeling have developed (23, 28, 29). We therefore propose designating the period from confirmed macrolide unresponsiveness to approximately 72 h thereafter as the “golden window” for bronchoscopic intervention. Acting within this timeframe maximizes clearance of mucus plugs and inflammatory mediators while preventing the cast consolidation of plastic bronchitis and the initiation of obliterative bronchiolitis that follow delayed management.

Capturing this golden window requires a reliable biomarker-assisted decision system (23). Clinical evidence suggests that combined, dynamic monitoring of serum lactate dehydrogenase, ferritin, and D-dimer offers particular value in predicting endoluminal plastic lesions (28–30). Elevated lactate dehydrogenase reflects the extent of cellular injury and necrosis; markedly raised ferritin signals excessive macrophage activation and an ongoing inflammatory storm; and progressively increasing D-dimer directly indicates the degree of fibrinous exudation and local coagulation cascade activation within the airways (28–30). The synergistic elevation of these three parameters forms a biomarker profile predictive of a high risk for bronchoscopically confirmed plastic bronchitis, providing an objective, quantifiable basis for ultra-early intervention that surpasses empirical judgment reliant on fever duration alone (29).

It should be emphasized that none of these biomarkers is specific to macrolide-unresponsive MPP when interpreted in isolation (10, 30, 31). Rather than serving as disease-specific diagnostic markers, their principal value lies in capturing complementary pathophysiological processes underlying disease progression, including tissue injury, hyperinflammation, and fibrin-rich airway remodeling (10, 32). Accordingly, the proposed biomarker triad is intended as a dynamic risk-stratification framework that should be interpreted in conjunction with clinical evolution and imaging findings, rather than as an independent criterion for determining bronchoscopic intervention. This multimodal approach is expected to improve patient selection while minimizing unnecessary invasive procedures in children with transient or self-limiting disease.

Bedside lung ultrasound has also assumed an increasingly recognized role in the early detection of high-risk features (33). The disappearance of dynamic air bronchograms—where intrabronchial gas shadows within consolidated lung are replaced by fluid-filled anechoic areas—indicates that the airway lumen has been filled with mucus plugs or fibrinous exudates (23, 34). This sonographic sign frequently emerges before the classic “finger-in-glove” sign or lobar atelectasis becomes apparent on computed tomography (CT) and can be monitored in real time at the bedside, offering an immediate, non-invasive imaging rationale for interventional decision-making (33, 34).

### 3.2 Modular combination strategy for interventional techniques based on immediate endoscopic phenotyping

Bronchoscopic intervention should not follow a standardized, uniform procedure but rather be assembled in a modular fashion according to the airway phenotype directly visualized (23, 35) (Table 1). For the inflammation-edema-dominant phenotype, characterized by severe mucosal hyperemia and edema with diffuse luminal narrowing but no prominent mucus plugs, the core strategy combines retention lavage with precisely targeted local corticosteroid perfusion, strictly limiting suction pressure to avoid mechanical trauma to the fragile, edematous mucosa (23). For the mucus-plug-dominant phenotype, in which viscous secretions fill the distal bronchi beyond the fourth generation, distal negative-pressure suction is combined with segmental lavage using mucolytics such as acetylcysteine or ambroxol to first loosen adherent secretions before their sequential aspiration (23, 28). For the plastic bronchitis phenotype, where fibrinous casts conforming to the bronchial architecture are visualized, the traditional “gentle-grasping” technique with biopsy forceps and the emerging cryoprobe “cryoadhesion” extraction technique each present distinct advantages and limitations (23, 28, 36). The cryoprobe method, which removes casts en bloc through freeze-adhesion, reduces fragment retention and minimizes mural abrasion (36). Preliminary evidence suggests its potential superiority under the principle of minimizing iatrogenic injury, although further comparative studies are needed (23, 36).

**TABLE 1:** Phenotype-guided modular bronchoscopic intervention strategies in MUMPP.

Endoscopic phenotype	Key features	Pathophysiology	Recommended intervention	Major precaution
Inflammation-edema dominant	Hyperemia, edema	Immune storm	Retention lavage + local corticosteroid perfusion	Avoid excessive suction
Mucus-plug dominant	Distal viscous plugs	Obstructive ventilation	Mucolytic lavage + distal suction	Prevent mucosal trauma
Plastic bronchitis phenotype	Fibrinous casts	Airway cast formation	Cryoadhesion/forceps extraction	Avoid cast fragmentation

MUMPP, macrolide-unresponsive *Mycoplasma pneumoniae* pneumonia.

### 3.3 A new ecosystem of peri-interventional management: coupling immunomodulation with respiratory mechanics protection

The bronchoscopic procedure itself constitutes only one component of the treatment continuum; cohesive peri-interventional management is equally essential (37). Early post-interventional anti-inflammatory bridging refers to the timely initiation of systemic corticosteroid therapy after bronchoscopic clearance of the inflammatory mediator reservoir (38). At this stage, corticosteroids can act upon a “cleansed” airway microenvironment, achieving stronger anti-inflammatory and anti-fibrotic effects (39). For respiratory mechanics monitoring, post-interventional individualized PEEP titration guided by transpulmonary pressure measurement or electrical impedance tomography can prevent bronchiole shear injury caused by cyclic alveolar collapse and recruitment, thereby consolidating the structural protection gained through the intervention (40). The concept of ciliary functional rehabilitation involves the sequential application of airway clearance techniques—such as high-frequency chest wall oscillation and postural drainage—during the post-interventional phase (41). These techniques support the gradual recovery of mucociliary clearance function, reduce the rate of re-obstruction, and complete a full therapeutic circuit from acute interventional bronchoscopy to functional airway restoration (41).

## 4 Practical challenges in strategy implementation and bottlenecks in translational research

### 4.1 Current state of evidence: bridging the gap from experience-driven to evidence-based practice

The clinical evidence supporting bronchoscopic intervention in MUMPP comes chiefly from single-center, retrospective case series (42). These studies share common methodological weaknesses: limited sample sizes, heterogeneous inclusion criteria, and imprecise descriptions of intervention timing and technique (42). More critically, the lack of standardized operating protocols across centers—covering lavage fluid composition, suction pressure parameters, adjunctive pharmacotherapy, and criteria for endoscopic phenotyping—makes inter-study comparison and quantitative synthesis extremely difficult (10, 42). Because of this homogeneity of limitations, clinical decision-making remains heavily dependent on individual operator experience and institutional custom, leaving a wide gap between current practice and genuinely evidence-driven care (10) (Table 2). Although the proposed framework is supported by a compelling pathophysiological rationale, the available evidence remains insufficient to support routine clinical implementation (43, 44). At present, it should therefore be regarded as a hypothesis-generating conceptual framework rather than a practice-changing clinical strategy. Future multicenter prospective studies adopting standardized protocols for patient selection, procedural techniques, peri-interventional nursing management, and long-term outcome evaluation will be essential to establish reproducible evidence across diverse clinical settings and facilitate responsible translation into routine practice.

**TABLE 2:** Current translational bottlenecks and future research priorities for bronchoscopic intervention in MUMPP.

Domain	Current limitation	Clinical impact	Future direction
Evidence quality	Retrospective single-center studies	Low evidence strength	Prospective multicenter trials
Timing standardization	No unified intervention criteria	Delayed intervention	Biomarker-based algorithms
Safety boundaries	Undefined pediatric thresholds	Iatrogenic injury risk	Age-stratified protocols
Outcome assessment	Reliance on fever/hospital stay	Misses long-term airway injury	Functional airway endpoints
Health economics	No cost-effectiveness models	Poor policy scalability	Economic modeling

MUMPP, macrolide-unresponsive *Mycoplasma pneumoniae* pneumonia.

The choice of efficacy endpoints introduces further bias. Most existing studies use defervescence time or length of hospital stay as primary outcome measures (45). Although easily obtained, these endpoints do not capture the intervention’s effect on the core pathological trajectory. The most serious long-term sequela of this disease is small airway dysfunction from obliterative bronchiolitis, which requires long-term follow-up through pulmonary function testing, impulse oscillometry, or high-resolution CT (45, 46). Substituting short-term clinical signs for long-term functional preservation risks overestimating the intervention’s true benefit (45, 47). Incorporating hard endpoints centered on distal airway function preservation is therefore essential for elevating evidence quality and closing the evidence gap.

### 4.2 Key controversies and safety boundaries in clinical implementation

Implementing early interventional strategies in young children involves a distinctive benefit-risk calculus (48). The narrow airway caliber and mucosal fragility of infants and young children lower the threshold for instrumentation-related mechanical injury compared with older patients (48). At the same time, repeated or prolonged general anesthesia and airway procedures may impose additional physiological burden and potential neurodevelopmental risk (49, 50). Establishing age-stratified safety boundaries that maximize lesional clearance while minimizing iatrogenic harm remains an unresolved central controversy.

Importantly, procedural risk should not be regarded as uniform across the pediatric population (51, 52). Future clinical protocols should therefore adopt age-adapted decision algorithms that integrate developmental physiology, disease severity, and anticipated procedural risk into individualized treatment planning. Children younger than 3 years may require a higher threshold for intervention because of increased susceptibility to anesthesia-related adverse effects and airway injury during critical stages of physiological and neurodevelopmental maturation (52). In this subgroup, bronchoscopy should be considered only when multiple objective indicators collectively suggest progressive airway compromise, whereas older children with greater physiological reserve may safely undergo earlier intervention when evidence supports evolving plastic bronchitis (14, 52, 53). Such an age-stratified framework may reduce unnecessary invasive procedures while preserving timely treatment for those most likely to benefit.

Beyond minimizing immediate procedural complications, future safety evaluation frameworks should adopt a broader longitudinal perspective. Comprehensive assessment should incorporate recovery of distal airway function, neurodevelopmental outcomes, and health-related quality of life to better characterize the overall impact of bronchoscopic intervention throughout childhood (54–56). Integrating these multidimensional safety endpoints into prospective pediatric studies will be essential for defining the long-term risk–benefit profile of early bronchoscopic intervention and facilitating its evidence-based clinical implementation.

Local corticosteroid perfusion presents further challenges. Although bronchoscopically delivered corticosteroids can rapidly suppress local inflammation, the optimal dosage, concentration, and dosing frequency remain undefined (57). Excessive or too frequent instillation may disproportionately suppress local immune surveillance and, especially given the already compromised epithelial barrier after mycoplasma infection, increase the risk of secondary bacterial or fungal infection (58). Pharmacokinetic studies and prospective clinical trials are urgently needed to define the balance between anti-inflammatory efficacy and immunosuppressive risk.

The potential for over-intervention also warrants careful consideration. Not all cases of macrolide-unresponsive pneumonia progress to severe plastic bronchitis or obliterative bronchiolitis (11, 59). Some children achieve complete resolution without invasive procedures despite transient fever and radiographic progression (60). Refining risk stratification criteria to reliably exclude potentially self-limiting cases from unnecessary invasive procedures is a pressing challenge for safeguarding patients against overtreatment.

### 4.3 Health economics and scalability considerations

Widespread dissemination of bronchoscopic interventional strategies faces significant barriers to technical penetration. In primary and secondary care settings, pediatric bronchoscopy equipment remains scarce, and there is a critical shortage of operators credentialed in pediatric airway procedures (61). Moreover, the capacity for rapid on-site evaluation—such as immediate cytological assessment to differentiate inflammatory phenotypes—is even more deficient at these levels of care (61, 62). These systemic barriers fundamentally constrain both the accessibility and the homogeneity of the strategy beyond large pediatric referral centers.

Finally, despite the theoretical potential of early intervention to shorten hospitalization and reduce long-term sequelae-related medical expenditure, formal health economic evaluations and cost-effectiveness analyses are entirely lacking (63, 64). The economic trade-off between the procedural costs of equipment, personnel, and anesthesia on the one hand, and the potentially avoided costs of chronic respiratory disease management on the other, has yet to be subjected to quantitative modeling (63). Addressing this gap in health economics evidence is a prerequisite for incorporating the strategy into health policy and standardized clinical pathways.

## 5 Summary

This perspective article consolidates the strategic triad of “timing priority, phenotype-guided intervention, and minimal-trauma principles” as the conceptual core for advancing bronchoscopic management of MUMPP. We contend that the therapeutic emphasis should shift decisively from delayed, salvage-oriented mucus evacuation toward early, precisely timed intervention within the golden window, guided by real-time endoscopic phenotyping and executed with techniques that prioritize mucosal preservation over procedural thoroughness alone.

Translating this framework into clinical reality demands the construction of a multidimensional early-warning model. Such a model should integrate clinical trajectory, the kinetics of lactate dehydrogenase, ferritin, and D-dimer, together with sonographic and CT imaging features, to objectively identify those children most likely to benefit from bronchoscopic intervention. By combining these complementary sources of information, clinical decision-making may progressively evolve from empirical judgment toward biomarker-informed, reproducible, and individualized intervention strategies.

Importantly, the present framework is intended to provide a precision-oriented conceptual foundation for future clinical investigation rather than to serve as an evidence-based standard of care. Its clinical implementation will require rigorous validation through prospective multicenter studies incorporating standardized intervention protocols, harmonized data collection, comprehensive safety evaluation, and long-term functional outcome assessment. Such evidence will be essential to determine whether this proposed paradigm can ultimately be translated into routine pediatric practice.

## Data Availability

The original contributions presented in this study are included in this article/supplementary material, further inquiries can be directed to the corresponding author.
